# Exosomal miR‐let‐7c‐5p is involved in the cognitive function of type 2 diabetes mellitus patients by interleukin 10: A cross‐sectional study

**DOI:** 10.1111/1753-0407.13450

**Published:** 2023-08-02

**Authors:** Hui Zhang, Shufang Yang, Wenwen Zhu, Tong Niu, Jue Wang, Mingyue Yang, Enlin Liu, Jumei Wang, Sumei Li, Haoqiang Zhang

**Affiliations:** ^1^ Henan Key Laboratory of Rare Diseases, Endocrinology and Metabolism Center The First Affiliated Hospital, and College of Clinical Medicine of Henan University of Science and Technology Luoyang China; ^2^ Department of Endocrinology Taizhou People's Hospital Taizhou China; ^3^ Department of Endocrinology Affiliated Zhongda Hospital of Southeast University Nanjing China; ^4^ Medical School Dalian Medical University Dalian China; ^5^ Medical School Nantong University Nantong China; ^6^ Department of Endocrinology The First Affiliated Hospital of USTC, Division of Life Sciences and Medicine, University of Science and Technology of China Hefei China

**Keywords:** exosome, interleukin 10, mild cognitve impairment, miR‐let‐7c‐5p, type 2 diabetes mellitus, 2型糖尿病, 炎症, 白细胞介素10, 外泌体, miR‐let‐7c‐5p

## Abstract

**Background:**

Interleukin (IL)‐10 plays a notable role in the inflammatory‐associated mild cognitive impairment (MCI). We aimed to investigate whether IL‐10 and its upstream factors exert an impact on MCI in type 2 diabetes mellitus (T2DM) patients.

**Methods:**

A total of 117 T2DM patients were recruited and divided into Control group and MCI group based on the presence or absence of MCI. Clinical parameters were collected. The Montreal Cognitive Assessment (MoCA) was conducted for global cognitive function. Digit Span Test (DST), Verbal Fluency Test (VFT), and Trail Making Test‐B (TMTB) were used to evaluate the executive functions of the diabetic patients. Trail Making Test‐A (TMTA) was performed to examine the information processing speed function. Patients' scene memory was examined by Logical Memory Test (LMT). After the baseline data were compared, correlation and regression analyses were performed to explore the relationship among IL‐10, miR‐let‐7c‐5p and cognitive function.

**Results:**

Compared to 80 patients in the control group, 37 patients in the MCI group exhibited lower IL‐10 in plasma and higher miR‐let‐7c‐5p levels in exosomes from plasma. The IL‐10 level was negatively associated with MoCA. Likewise, miR‐let‐7c‐5p levels were negatively correlated with IL‐10 levels and MoCA. Elevated miR‐let‐7c‐5p levels and decreased IL‐10 levels are risk factors for MCI in T2DM patients. Increased miR‐let‐7c‐5p and downregulated IL‐10 may influence VFT and TMTB, respectively, associated with executive function.

**Conclusions:**

We demonstrated that IL‐10 is correlated to the executive function of T2DM patients. Decreased IL‐10 may result from the regulation of miR‐let‐7c‐5p in exosomes.

## Graphical abstract

**Highlights**

- Both interleukin‐10 levels and exosomal miR‐let‐7c‐5p levels are correlated with cognitive function in patients with type 2 diabetes mellitus.
- There is a negative association between interleukin‐10 levels and exosomal miR‐let‐7c‐5p levels in the plasma of patients with type 2 diabetes mellitus.

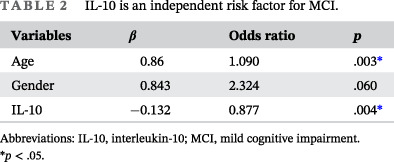

## 摘要

目的:白细胞介素(IL)‐10在与炎症相关的轻度认知障碍(MCI)中起着显著作用。我们旨在研究IL‐10及其上游因素是否对2型糖尿病(T2DM)患者的MCI产生影响。

方法:共招募117名T2DM患者, 根据是否存在MCI将其分为对照组和MCI组。收集临床参数。进行蒙特利尔认知评估(MoCA)以评估整体认知功能。使用数字跨度测试(DST)、言语流畅性测试(VFT)和连线测验B(TMTB)评估糖尿病患者的执行功能。进行连线测验A(TMTA)以检查信息处理速度功能。通过逻辑记忆测试(LMT)检查患者的场景记忆。比较基线数据后, 进行相关性和回归分析, 探讨IL‐10(miR‐let‐7c‐5p)与认知功能之间的关系。

结果:与80名对照组患者相比, 37名MCI组患者表现出较低的血浆IL‐10水平和较高的血浆外泌体中miR‐let‐7c‐5p水平。IL‐10水平与MoCA呈负相关。同样, miR‐let‐7c‐5p水平与IL‐10水平和MoCA呈负相关。升高的miR‐let‐7c‐5p水平和降低的IL‐10水平是T2DM患者MCI的危险因素。增加的miR‐let‐7c‐5p和下调的IL‐10可能分别影响与执行功能相关的VFT和TMTB。

结论:我们表明IL‐10与T2DM患者执行功能相关。降低的IL‐10可能是由于外泌体中miR‐let‐7c‐5p的调控所致。

## 1 INTRODUCTION

With the increased prevalence of diabetes in China^1^ and worldwide,^2^ its complications, including mild cognitive impairment (MCI), have been receiving increasing attention from researchers.^3^, ^4^ MCI is an early stage of dementia.^5^ Owing to the lack of clarified mechanisms and severe clinical symptoms, there are limited treatment methods and poor prognosis for dementia. Therefore, MCI is an intervenable stage between normal cognition and dementia.^6^

Neuroinflammation is one of the characteristics of diabetic cognitive impairment.^7^ Microglia activation mediated neuroinflammation plays a major role in cognitive dysfunction in diabetes.^8^, ^9^, ^10^ Interleukin‐10 (IL‐10) is involved in the activation of microglia. On the one hand, IL‐10 could inhibit the transformation of microglia to the M1 phenotype, which is a proinflammatory phenotype; on the other hand, it may inhibit the secretion of interleukin‐1β, interleukin‐6, tumor necrosis factor‐α, and other inflammatory factors from microglia.^11^

Exosomes are extracellular vesicles secreted by living cells, containing complex RNA and protein components, with a diameter of about 30–150 nm.^12^ They play an important role in intercellular dialog and participate in the occurrence and development of diabetes and its complications, especially diabetic neuropathy,^13^ which is related to the biological activity of its components, including microRNA.^14^ Indeed, several miR‐let‐7 family members could bind to the 3 ‘UTR end of IL‐10 mRNA, reducing IL‐10 transcription without affecting the stability of IL‐10 mRNA.^15^, ^16^ Additionally, miR‐let‐7c‐5p could suppress inflammation by inhibiting the dentin matrix protein‐1‐ mediated nuclear factor kappa B pathway in vitro and in vivo.^17^ Exosome‐derived miR‐let‐7c promotes angiogenesis in multiple myeloma by polarizing macrophages.^18^ Moreover, miR‐let‐7i‐5p is a promising serum biomarker for poststroke cognitive impairment and alleviated oxygen glucose deprivation‐induced cell damage in vitro by regulating Bcl‐2^19^ and involved in inflammation associated cells apoptosis.^20^

Here, we aim to investigate the relationship among exosomal miR‐let‐7c‐5p (or miR‐let‐7i‐5p), IL‐10 levels in plasma, and MCI in type 2 diabetes mellitus (T2DM) patients via the present cross‐sectional research.

## 2 METHODS

### 2.1 Experimental design and ethics

All patients were recruited from the Department of Endocrinology, Taizhou People's Hospital. IL‐10, miR‐let‐7c‐5p, and miR‐let‐7i‐5p measurement were conducted at the First Affiliated Hospital of the University of Science and Technology of China. These 117 patients were certificated by the standard of T2DM. Of these patients, 37 T2DM individuals were diagnosed as MCI, and 80 individuals exhibited a healthy cognitive function. All participants were informed about this study and signed the informed consent agreement. This cross‐sectional study was approved by the Ethics Committee for Medical Research, Taizhou People's Hospital (Approval No.: 202204601). The experiment was performed from April 2022 to December 2022.

### 2.2 Inclusion and exclusion criteria

The inclusion criteria were described as follows. Inpatients (with T2DM duration ≥3 years) were recruited for this study. All patients with T2DM met the World Health Organization 1999 criteria.^21^ Thirty‐seven patients with MCI satisfied the criteria proposed by the MCI Working Group of the European Consortium on Alzheimer's disease.^22^ The subjects for exclusion were the similar with our previous study^23^ and specifically described as follows: (a) recent diagnosed acute complications of diabetes; (b) severe low plasma glucose; (c) acute vascular disease of heart and brain; (d) alcohol or drug abuse; (e) diagnosed disease of thyroid (with thyroid dysfunction or abnormal autoimmune antibodies); (f) severe infection or major surgery; (g) visual or hearing dysfunction (cannot finish neuropsychological tests); (h) dementia (severe cognitive decline out of the range of MCI); (i) other diseases that may affect (or potentially influence) cognition; and (j) cognitive function testing and inflammation, like anemia, cancer, and autoimmune disease (e.g., Crohn's disease, rheumatoid arthritis, systemic lupus erythematosus, and so on). Patients were divided into Control group and MCI group based on the absence or presence of MCI, respectively.

### 2.3 Clinical data collection and neuropsychological tests

The information of age, gender, and the duration of education and DM of all patients was collected. Weight (kg)/height (m)^2^ was used to calculate the body mass index (BMI). Triglyceride (TG; Roche Group, Basel, Switzerland; detection range: 0.1–10.0 mmol/L), total cholesterol (TC; Roche Group, Basel, Switzerland; detection range: 0.1–20.7 mmol/L), high‐density lipoprotein cholesterol (HDL‐C; Roche Group, Basel, Switzerland; detection range: 0.08–3.88 mmol/L), and low‐density lipoprotein cholesterol (LDL‐C; Ningbo Ruiyuan Biotechnology Co., Ltd., Ningbo, China; detection range: 0.2–11.6 mmol/L) in blood samples were measured at the Center Laboratory of Taizhou People's Hospital, Taizhou. As part of the multicenter research, neuropsychological tests were conducted in accordance with the same method described previously.^23^

### 2.4 Enzyme‐linked immunosorbent assay

Blood samples were freshly collected on the second day after the patients were hospitalized. The collected blood samples were centrifuged at 1000*g* (30 min, 4°C) to obtain plasma. IL‐10 in plasma was determined by ELISA in accordance with the instructions from the manufacturer (Cloud‐Clone Crop., Wuhan, China, Catalogue No.: SEA056Hu). This kit has a detection range of 7.8–500 pg/mL, an intra‐assay value of CV < 10%, and an interassay value of CV < 12%.

### 2.5 Exomonal micro‐RNA isolation and measurement

Plasmatic exosomes were isolated from blood by using the exoRNeasy Kits (QIAGEN, Shanghai, China, Catalogue No.: 77144) in accordance with the protocol of the manufacturer. The relative expression levels of miR‐let‐7c‐5p and miR‐let‐7i‐5p were normalized to U6. The primers (forward and reverse) were as follows: miR‐let‐7c‐5p: 5′‐CGTCATCCTGAGGTAGTAGGTTGT‐3′ and 5′‐TATGGTTTTGACGACTGTGTGAT‐3′; miR‐let‐7i‐5p: 5′‐CGGGCTGAGGTAGTAGTTTG‐3′ and 5′‐CAGCCACAAAAGAGCACAAT‐3′; U6: 5′‐CAGCACATATACTAAAATTGGAACG‐3′ and 5′‐ACGAATTTGCGTGTCATCC‐3′. Total microRNA was extracted from exosomes by Exosomal RNA Isolation Kit (Norgen Biotek Corp., Ontario, Canada, Catalogue No.: 58000) in accordance with the instruction of the manufacturer. The RNA concentration was measured on the basis of A260/280 absorbance by NanoDrop2000. The extracted microRNA was reverse transcribed to cDNA by using an miRNA 1st Strand cDNA Synthesis Kit (Vazyme, Nanjing, China, Catalogue No.: MR201‐02). Real‐time polymerase chain reaction was performed by using an miRNA Universal SYBR qPCR Master Mix (Vazyme, Nanjing, China, Catalogue No.: MQ101‐02) in accordance with the protocol of the manufacturer.

### 2.6 Bioinformatic analysis

TargetScan (www.targetscan.org) was used to predict the combination site between miR‐let‐7c‐5p and IL‐10 mRNA.

### 2.7 Sample size calculation and statistical methods

The minimum sample size for this study was calculated using PASS V11.0.7 (NCSS, USA). We estimated the minimum sample size by the mean and SD of IL‐10 as we finished part of the volunteer recruitment work. When we completed all of the volunteer recruitment work, we confirmed that the sample size was sufficient. The minimum sample sizes of individuals in the control group and those in the MCI group are 74 and 35, respectively. Data were analyzed using SPSS 22.0 (IBM, USA). Student's *t* test, nonparametric Mann–Whitney *U* test, and *χ* ^2^ test were carried out for normally distributed variables, asymmetrically distributed variables, and binary variables, respectively. Pearson and partial correlation analyses, as well as binary logistic analyses, and multiple linear regression were performed to investigate the association between IL‐10 (or miR‐let‐7c‐5p) and MCI. A *p* < .05 was considered as statistically significant.

## 3 RESULTS

### 3.1 Clinical parameters of patients with T2DM

To explore the potential risk factors of MCI of patients with T2DM, the clinical data of diabetic patients with MCI and those without MCI were compared. As shown in Table 1, we did not observe significant difference in the duration of DM and education, BMI, glycated hemoglobin (HbA1c), C peptide, TG, TC, LDL‐C, and HDL‐C levels in T2DM patients with MCI and those with normal cognitive function (all indicate *p* > .05). However, patients with cognitive decline are older than those without MCI (*p* = .002). Additionally, compared to patients in the control group, there are more female in the MCI group (*p* = .026). Interestingly, the IL‐10 levels are higher in diabetic patients with healthy cognition than in those with MCI (*p* = 0.06) (Table 1).

**TABLE 1:** Comparation of clinical parameters and IL‐10 levels between control and MCI group.

Variables	Control (*n* = 80)	MCI (*n* = 37)	*p*
Age (years)	53.50 (45.25–58.00)	59.00 (53.00–65.00)	.002^b^_,_ *
Female (*n*, %)	24–30.00	19–51.35	.026^c^_,_ *
Education (year)	12.00 (9.00–15.00)	12.00 (6.00–12.00)	.055^b^
DM duration (year)	9.00 (5.00–13.00)	9.00 (5.00–13.50)	.702^b^
BMI (Kg/m^2^)	25.01 (22.39–27.49)	24.24 (22.55–26.39)	.462^b^
HbA1c (%)	8.83 ± 1.96	8.75 ± 1.34	.792^a^
FPG (mmol/L)	7.67 (6.02–10.22)	7.57 (5.92–8.97)	.290^b^
C peptide (pmol/L)	517.97 (280.20–693.50)	414.73 (290.06–597.39)	.238^b^
TG (mmol/L)	1.46 (1.03–2.42)	1.49 (0.80–2.43)	.824^b^
TC (mmol/L)	4.03 (3.56–4.83)	4.35 (3.37–5.38)	.534^b^
HDL‐C (mmol/L)	0.97 (0.84–1.16)	1.01 (0.88–1.19)	.476^b^
LDL‐C (mmol/L)	2.58 (2.24–3.06)	2.73 (2.18–3.19)	.656^b^
MoCA	28.00 (27.00–28.00)	23.00 (21.00–25.00)	.000^b^_,_ *
DST	12.00 (10.00–13.00)	11.00 (9.00–12.00)	.005^b^_,_ *
VFT	18.00 (16.00–21.00)	14.00 (12.50–17.00)	.000^b^_,_ *
TMTA	53.00 (40.25–67.25)	75.00 (51.50–98.00)	.000^b^_,_ *
TMTB	132.50 (100.25–167.75)	178.00 (136.50–249.50)	.000^b^_,_ *
LMT	9.00 (6.00–13.00)	8.00 (4.50–12.00)	.100^b^
IL‐10 (pmol/L)	88.79 ± 5.51	85.84 ± 4.87	.006^a^_,_ *

Abbreviations: BMI, body mass index; DM, diabetes mellitus; DST, Digit Span Test; FPG, fasting plasma glucose; HDL‐C, high density lipoprotein cholesterol; IL‐10, interleukin‐10; LDL‐C, low density lipoprotein cholesterol; LMT, Logical Memory Test; MCI, mild cognitive impairment; MoCA, Montreal Cognitive Assessment; TC, total cholesterol; TG, triglycerides; TMTA, Trail Making Test‐A; TMTB, Trail Making Test‐B; VFT, Verbal Fluency Test.

^a^ Student's *t* test was employed for normally distributed variables.

^b^ The Mann–Whitney *U* test was employed for asymmetrically distributed variables.

^c^ The *χ* ^2^‐test was employed for categorical variables.

* *p* < .05.

### 3.2 Pearson correlation between IL‐10 and cognitive function

**FIGURE 1:**
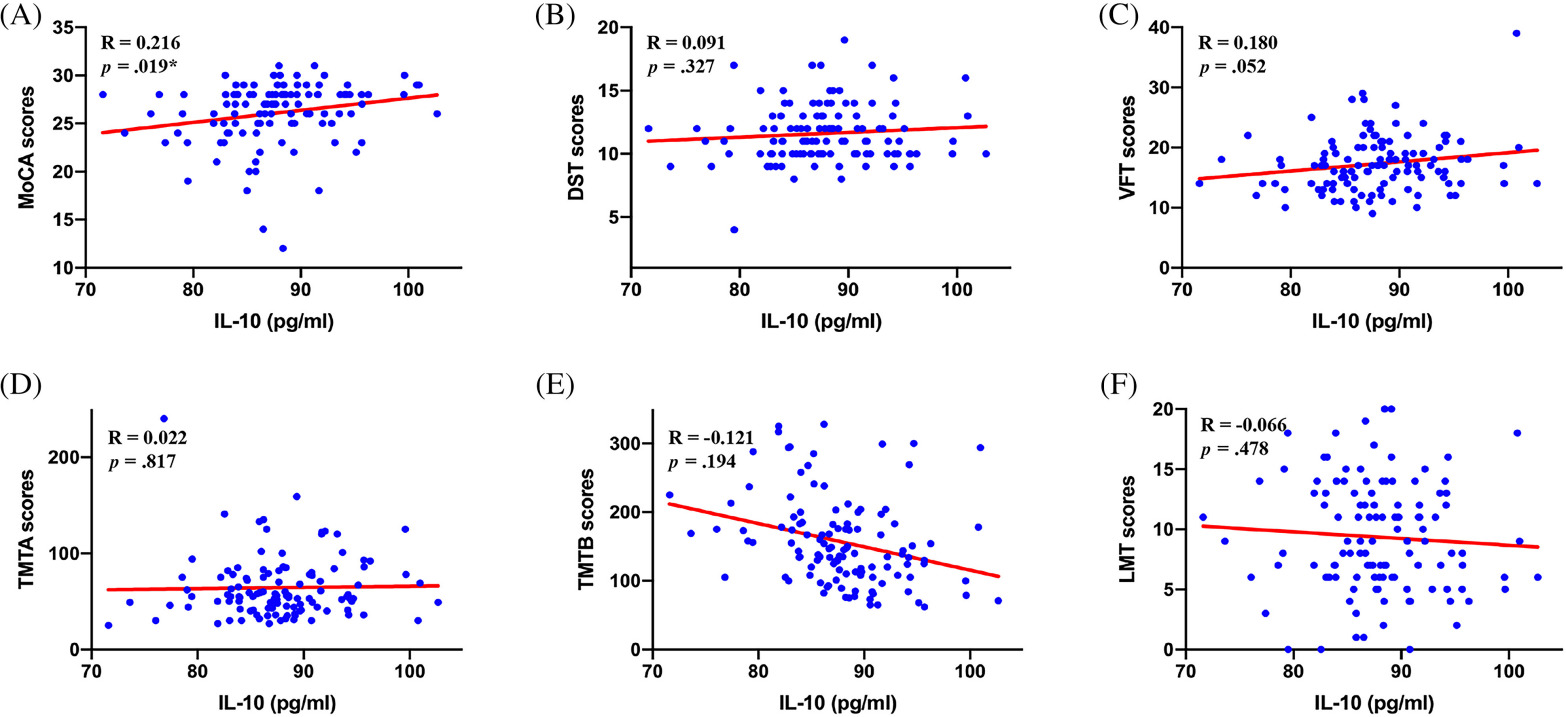
Association between IL‐10 and cognitive function. *p < .05; (A) showed a positive association between exosomal IL‐10 levels and MoCA scores in patients with T2DM; (B) did not show a significant association between exosomal IL‐10 levels and DST scores in patients with T2DM; (C) did not show a significant association between exosomal IL‐10 levels and VFT scores in patients with T2DM; (D) did not show a significant association between exosomal IL‐10 levels and TMTA scores in patients with T2DM; (E) did not show a significant association between exosomal IL‐10 levels and TMTB scores in patients with T2DM; (F) did not show a significant association between exosomal IL‐10 levels and LMT scores in patients with T2DM. DST, Digit Span Test; IL‐10, interleukin‐10; LMT, Logical Memory Test; MoCA, Montreal Cognitive Assessment; T2DM, type 2 diabetes mellitus; TMTA, Trail Making Test‐A; TMTB, Trail Making Test‐B; VFT, Verbal Fluency Test.

To investigate the relationship between IL‐10 and diabetic cognitive dysfunction, the Pearson correlation analysis was conducted. We found that IL‐10 levels are positively associated with Montreal Cognitive Assessment (MoCA) scores (*R* = 0.216, *p* = 0.019) but not related to Digit Span Test (DST), Trail Making Test‐A (TMTA), Trail Making Test‐A (TMTB), and Logical Memory Test (LMT) scores (all *p* > .05). Although no statistical significance was found, the IL‐10 levels seem to be associated with Verbal Fluency Test (VFT) scores (*R* = 0.180, *p* = .052) (Figure 1).

### 3.3 Partial correlation between IL‐10 levels and cognitive function adjusted for age and gender

Given the age and gender difference of T2DM patients with and without MCI, a partial correlation analysis was conducted to adjust for these two factors. We found not only a positive association between IL‐10 and MoCA scores (*R* = 0.242, *p* = .009) but also a positive correlation between IL‐10 and VFT scores (*R* = 0.200, *p* = .032) (Table S1).

### 3.4 Low IL‐10 level is one of the risk factors of MCI in patients with T2DM

For further exploration of the notable role of IL‐10 in T2DM patients with MCI, binary logistic regression was conducted and adjusted by age and gender. The result showed that a lower IL‐10 level is one of the risk factors of MCI, independent of age and gender in patients with T2DM (*p* = .004) (Table 2).

**TABLE 2:** IL‐10 is an independent risk factor for MCI.

Variables	*β*	Odds ratio	*p*
Age	0.86	1.090	.003*
Gender	0.843	2.324	.060
IL‐10	−0.132	0.877	.004*

Abbreviations: IL‐10, interleukin‐10; MCI, mild cognitive impairment.

* *p* < .05.

### 3.5 Low IL‐10 levels may influence the VFT scores of T2DM patients

To clarify the effect of IL‐10 levels on the details of cognitive function in patients with T2DM, multiple linear regression was conducted. After the adjustment by age and gender, the result demonstrated that low IL‐10 may influence the VFT scores of patients with T2DM (*β* = 0.052, *p* = .032) (Table S2).

### 3.6 Comparison of miR‐let‐7c‐5p and miR‐let‐7i‐5p levels in different patients with T2DM

Compared to the T2DM patients without MCI, the T2DM patients with MCI showed increased miR‐let‐7c‐5p levels (*p* = .007) but not miR‐let‐7i‐5p levels (*p* = .124) of exosomes from the patients' plasma (Figure S1A,B).

### 3.7 Association between IL‐10 and miR‐let‐7c‐5p levels in patients with T2DM

To explore the relationship between IL‐10 and miR‐let‐7c‐5p levels, the Pearson correlation analysis was carried out. Indeed, there was a significant association between IL‐10 and miR‐let‐7c‐5p in patients with T2DM (*R* = −0.018, *p* = .046) (Figure S1C). Bioinformatic analysis was also performed to explore the combination site between miR‐let‐7c‐5p and IL‐10 mRNA by using TargetScan. We found that miR‐let‐7c‐5p may bind to positions 140–146 of IL‐10 3′ UTR of IL‐10 mRNA (Figure S1D).

### 3.8 Pearson correlation between miR‐let‐7c‐5p and cognitive function

**FIGURE 2:**
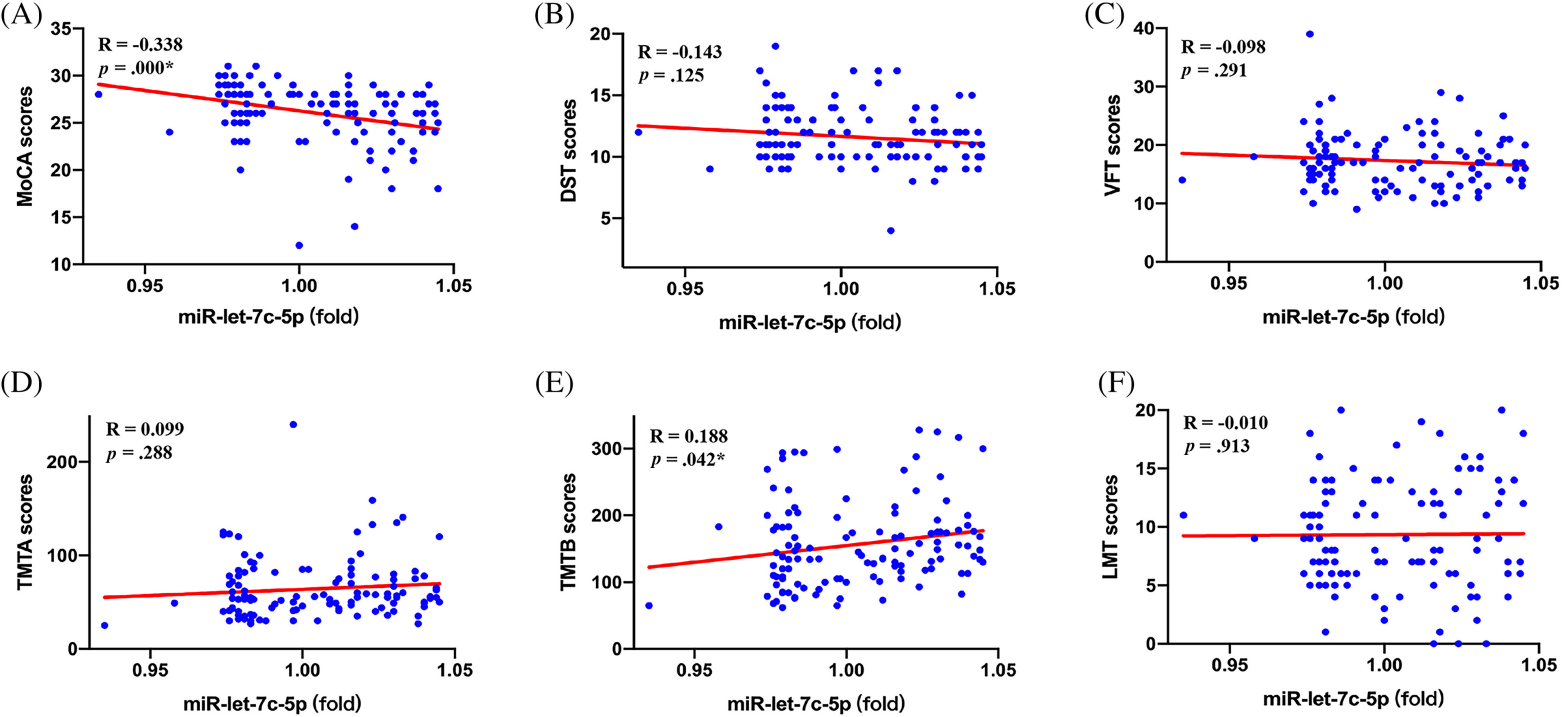
Association between miR‐let‐7c‐5p and cognitive function. *p < .05; (A) showed a negative association between exosomal miR‐let‐7c‐5p levels and MoCA scores in patients with T2DM; (B) did not show a significant association between exosomal miR‐let‐7c‐5p levels and DST scores in patients with T2DM; (C) did not show a significant association between exosomal miR‐let‐7c‐5p levels and VFT scores in patients with T2DM; (D) did not show a significant association between exosomal miR‐let‐7c‐5p levels and TMTA scores in patients with T2DM; (E) showed a positive association between exosomal miR‐let‐7c‐5p levels and TMTB scores in patients with T2DM; (F) did not show a significant association between exosomal miR‐let‐7c‐5p levels and LMT scores in patients with T2DM. DST, Digit Span Test; IL‐10, interleukin‐10; LMT, Logical Memory Test; MoCA, Montreal Cognitive Assessment; T2DM, type 2 diabetes mellitus; TMTA, Trail Making Test‐A; TMTB, Trail Making Test‐B; VFT, Verbal Fluency Test.

To clarify the relationship between miR‐let‐7c‐5p and diabetic cognitive dysfunction, the Pearson correlation analysis was conducted. We found that miR‐let‐7c‐5p levels are associated with MoCA scores negatively (*R* = −0.328, *p* = .000) and associated with TMTB scores positively (*R* = 0.188, *p* = .042), but they are not related to DST, TMTA, and LMT scores (all indicate *p* > .05) (Figure 2).

### 3.9 Partial correlation between miR‐let‐7c‐5p levels and cognitive function adjusted for age and gender

Given the age and gender difference of T2DM patients with and without MCI, a partial correlation analysis was conducted to adjust for these two factors. We found a negative association between miR‐let‐7c‐5p and MoCA (*R* = −0.282, *p* = .002) but did not observe a correlation between IL‐10 and TMTB (*R* = 0.085, *p* = .366) (Table S3).

### 3.10 Increased miR‐let‐7c‐5p level was one of the risk factors of MCI in patients with T2DM

To investigate the role of miR‐let‐7c‐5p in diabetic patients with MCI, binary logistic regression was conducted, adjusted by age and gender. The result showed that increased miR‐let‐7c‐5p level is one of the risk factors of MCI, independent from age and gender in patients with T2DM (*p* = .035) (Table 3).

**TABLE 3:** miR‐let‐7c‐5p is an independent risk factor for MCI.

Variables	*β*	Odds ratio	*p*
Age	0.064	1.066	.014*
Gender	0.795	2.214	.069
miR‐let‐7c‐5p	19.381	261 323 800	.035*

Abbreviation: MCI, mild cognitive impairment.

* *p* < .05.

## 4 DISCUSSION

To explore the possible mechanism of diabetic cognitive impairment in T2DM patients, we recruited 117 patients with or without MCI in this work. We compared the baseline data of patients in the control group and the MCI group. Given that this work is a cross‐sectional study, the data of age and gender are not well matched in these two groups. Thus, these two factors were adjusted in the following research. In addition to the comparison of the clinical characteristics of T2DM patients with and without MCI, their neuropsychological preference was also compared. Decreased MoCA, DST, and VFT scores as well as elevated TMTA/TMTB scores were found in diabetic patients with MCI. The lower MoCA scores in diabetic patients with MCI than those in patients without MCI confirmed the global cognitive impairment.^24^ Decreased DST^25^ and VFT^26^ and increased TMTB scores showed impaired executive function. The elevated TMTA scores exhibited the dysfunction of information processing speed ability.^27^

As described in the Introduction, microglia activation‐mediated neuroinflammation plays an important role in cognitive dysfunction in diabetes.^8^, ^9^, ^10^ Additionally, IL‐10 could inhibit the kind of neuroinflammation medicated by microglia.^11^ Hence, the levels of IL‐10 in T2DM patients with normal cognition and cognitive dysfunction were compared. Interestingly, there is a suppressed IL‐10 level in MCI patients with T2DM, compared to those diabetic patients with healthy cognition. This result agrees with the result of a study that showed increased IL‐10 levels and improved episodic memory after 12 months of anti‐Alzheimer's disease treatment.^28^ IL‐10 is not only associated with MoCA scores but is also related to VFT scores adjusted by age and gender in partial correlation analysis. The binary logistic regression demonstrated that lower IL‐10 is an independent risk factor of diabetic MCI in patients. Further multiple linear regression suggested that decreased IL‐10 may influence the scores of VFT, exhibiting the executive function. IL‐10 was demonstrated to be possibly involved in the MCI of T2DM patients, especially for the executive ability. Indeed, Yang et al demonstrated that cortisol is related to patients' executive function associated with IL‐10.^29^

This study analyzed the association between plasma IL‐10 and MCI, especially for executive function in T2DM patients. However, the possible mechanism between IL‐10 in plasma and MCI, which is associated with the main pathological changes in the central nervous system, needs further exploration. Exosomes are involved in the occurrence and development of diabetes and its complications, including diabetic neuropathy.^13^ They could easily pass through the blood–brain barrier and mediate the cross‐talk between the central nervous system and peripheral tissue. Moreover, the content, especially for noncoding RNA, in exosomes keeps their biological activity. Indeed, miR‐let‐7 family members could bind to 3′ UTR and affect the function of IL‐10 mRNA.^15^, ^16^ miR‐let‐7i‐5p and miR‐let‐7c‐5p are also associated with poststroke cognitive impairment and inflammation‐associated cell phenotype change, respectively. Here, the levels of miR‐let‐7i‐5p and miR‐let‐7c‐5p were measured. Although no significant difference in exosomal miR‐let‐7i‐5p was observed between diabetic patients with MCI and those without MCI, we found elevated exosomal miR‐let‐7c‐5p levels in the MCI group, compared with those in the control group. Actually, miR‐let‐7c in extracellular vesicles is a potential biomarker for early osteoarthritis,^30^ which is another inflammation‐associated disease,^31^ similar to diabetic cognition dysfunction.

To further explore the relationship between IL‐10 levels in plasma and plasma exosomal miR‐let‐7c‐5p levels, the association between them was analyzed. A negative correlation between plasmic IL‐10 and exosomal miR‐let‐7c‐5p was identified in patients with T2DM. A combination site exists between positions 140–146 of IL‐10 3′ UTR and miR‐let‐7c‐5p.

Aside from the relationship between miR‐let‐7c‐5p and IL‐10, we also analyzed the correlation between exosomal miR‐let‐7c‐5p in plasma and cognitive performance. Here, not only the positive correlation between miR‐let‐7c‐5p and MoCA scores but also the negative association between miR‐let‐7c‐5p and TMTB scores were detected by the Pearson correlation analysis. The exosomal miR‐let‐7c‐5p in plasma may also be associated with the cognitive function of T2DM patients. Furthermore, elevated exosomal miR‐let‐7c‐5p in plasma is one of the risk factors of MCI in T2DM patients, adjusted by age and gender.

## 5 CONCLUSION

Increased exosomal miR‐let‐7c‐5p may be involved in the MCI of patients with T2DM by downregulating IL‐10. On the one hand, increased exosomal miR‐let‐7c‐5p from the brain may be released to blood and regulate the levels of IL‐10 in peripheral tissues. On the other hand, miR‐let‐7c‐5p from peripheral tissues may affect the central nervous system by regulating the neuroinflammation via IL‐10. Both situations may exist at the same time.

### 5.1 Limitations

To our best knowledge, we were the first to investigate the relationship between MCI and IL‐10 associated with exosomal miR‐let‐7c‐5p in T2DM patients. However, some limitations need to be mentioned. First, we measured the levels of IL‐10 in plasma in this work. However, it cannot reflect the situation of inflammation in the central nervous system. Although IL‐10 is easier to be observed in plasma than in cerebrospinal fluid, the levels of IL‐10 in cerebrospinal fluid may better reflect the concentration of IL‐10 in the brain. Second, we isolated all exosomes from plasma for the cost. As a matter of fact, exosomes derived from microglia, neurons, or other specific cells from the central nervous system and peripheral tissues may be another choice. Third, well‐designed cohort studies are needed to further clarify the causal relationship between IL‐10 (or miR‐let‐7c‐5p), and diabetic cognition decline. Additionally, the relationship between IL‐10 and miR‐let‐7c‐5p needs to be further explored by basic experiments. Fourth, we have tried our best to set exclusion criteria to avoid other factors that may influence IL‐10 levels and confirm the important role of IL‐10 in diabetic cognitive impairment. However, as a clinical study, the mechanism investigation is far inferior to basic research.

## AUTHOR CONTRIBUTIONS

All authors made significant contributions to the work reported. Haoqiang Zhang and Sumei Li contributed to the idea. Hui Zhang wrote the manuscript draft. Shufang Yang revised the manuscript. Wenwen Zhu, Tong Niu, Jue Wang, Mingyue Yang, Enlin Liu, and Jumei Wang collected the data or performed the tests and/or statistical analysis. All authors gave final approval of the version to be published, agreed on the journal to which the article has been submitted, and agreed to be accountable for all aspects of the work.

## DISCLOSURE

The authors report no interest conflicts in this work.

## Supporting information


**Figure S1.** Analysis of the relationship between miR‐let‐7 members and IL‐10. Legend: *p < .05; (A) showed elevated miR‐let‐7c‐5p levels in T2DM patients with MCI, compared to those without MCI; (B) did not show significant difference of miR‐let‐7i‐5p between T2DM patients in MCI group and control group. (C) showed a negative association between IL‐10 levels and exosemal miR‐let‐7c‐5p levels in patients with T2DM. (D) showed the possible combination site between miR‐let‐7c‐5p and IL‐10 mRNA. IL‐10, interleukin‐10; MCI, mild cognitive impairment; T2DM, type 2 diabetes mellitus.

**Table S1.** Association between IL‐10 and MoCA (VFT) scores adjusted for age and gender. Abbreviations: IL‐10, interleukin‐10; MoCA, Montreal Cognitive Assessment; VFT, Verbal Fluency Test.
**Table S2.** Analysis for factors influence the VFT scores of T2DM patients. Abbreviations: T2DM, type 2 diabetes mellitus; VFT, Verbal Fluency Test.
**Table S3.** Association between miR‐let‐7c‐5p and MoCA (TMTB) scores adjusted for age and gender. Abbreviations: MoCA, Montreal Cognitive Assessment; TMTB, Trail Making Test‐B.

## Data Availability

All data in this manuscript have been submitted to The First Affiliated Hospital of USTC for records. The ID of recruited patients was also collected for further usage. All data are available on reasonable request from corresponding authors.
