# Supplementary figures and images for: Exosomal miR‐let‐7c‐5p is involved in the cognitive function of type 2 diabetes mellitus patients by interleukin 10: A cross‐sectional study
Source: J Diabetes. 2023 Aug 2;15(11):978–86. doi: 10.1111/1753-0407.13450 (PMC10667643; doi:10.1111/1753-0407.13450)

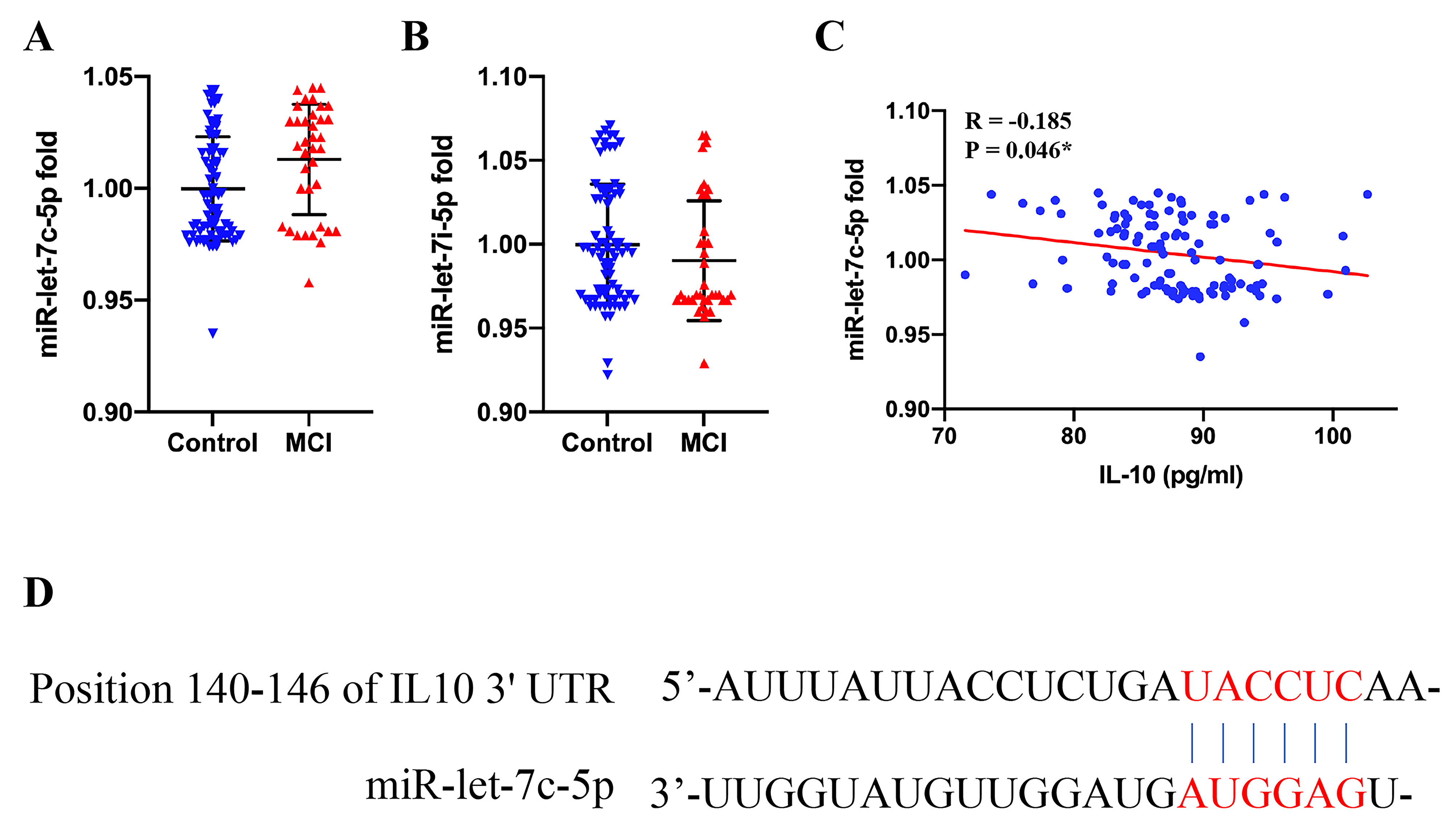

Supplement: Supplementary file 1 — Figure S1. Analysis of the relationship between miR‐let‐7 members and IL‐10. Legend: *p < .05; (A) showed elevated miR‐let‐7c‐5p levels in T2DM patients with MCI, compared to those without MCI; (B) did not show significant difference of miR‐let‐7i‐5p between T2DM patients in MCI group and control group. (C) showed a negative association between IL‐10 levels and exosemal miR‐let‐7c‐5p levels in patients with T2DM. (D) showed the possible combination site between miR‐let‐7c‐5p and IL‐10 mRNA. IL‐10, interleukin‐10; MCI, mild cognitive impairment; T2DM, type 2 diabetes mellitus. [file JDB-15-978-s002.tif]
