# Supplementary material for: Exosomal miR‐let‐7c‐5p is involved in the cognitive function of type 2 diabetes mellitus patients by interleukin 10: A cross‐sectional study
Source: J Diabetes. 2023 Aug 2;15(11):978–86. doi: 10.1111/1753-0407.13450 (PMC10667643; doi:10.1111/1753-0407.13450)
Supplement: Supplementary file 2 — Table S1. Association between IL‐10 and MoCA (VFT) scores adjusted for age and gender. Abbreviations: IL‐10, interleukin‐10; MoCA, Montreal Cognitive Assessment; VFT, Verbal Fluency Test. Table S2. Analysis for factors influence the VFT scores of T2DM patients. Abbreviations: T2DM, type 2 diabetes mellitus; VFT, Verbal Fluency Test. Table S3. Association between miR‐let‐7c‐5p and MoCA (TMTB) scores adjusted for age and gender. Abbreviations: MoCA, Montreal Cognitive Assessment; TMTB, Trail Making Test‐B. [file JDB-15-978-s001.docx]

Supplementary Table 1: Association between IL-10 and MoCA (VFT) scores adjusted for age and gender

|  | R | P |
| --- | --- | --- |
| MoCA | 0.242 | 0.009* |
| VFT | 0.200 | 0.032* |

Notes:

*P<0.05

Abbreviations: IL-10, interleukin-10; MoCA, Montreal Cognitive Assessment; VFT, Verbal Fluency Test.

Supplementary Table 2: Analysis for factors influence the VFT scores of T2DM patients

|  | β | 95% CL for β | | P |
| --- | --- | --- | --- | --- |
|  |  | lower | upper |  |
| Age | -0.156 | -0.235 | -0.077 | 0.000* |
| Gender | 1.376 | -0.255 | 3.007 | 0.097 |
| IL-10 | 0.157 | 0.014 | 0.301 | 0.032* |

Notes:

*P<0.05

Abbreviations: VFT, Verbal Fluency Test; IL-10, interleukin-10; T2DM, type 2 diabetes mellitus.

Supplementary Table 3: Association between miR-let-7c-5p and MoCA (TMTB) scores adjusted for age and gender

|  | R | P |
| --- | --- | --- |
| MoCA | -0.275 | 0.039* |
| TMTB | 0.062 | 0.507* |

Notes:

*P<0.05

Abbreviations: MoCA, Montreal Cognitive Assessment; TMTB, Trail Making Test-B;
